# A Retrospective Study of Urinary Schistosomiasis in the Eastern Cape Province, South Africa

**DOI:** 10.3390/tropicalmed9120293

**Published:** 2024-11-30

**Authors:** Dominic Targema Abaver

**Affiliations:** 1 HERENDA Program, New Medical School, Walter Sisulu University, Nelson Mandela Drive, Mthatha 5100, Eastern Cape, South Africa; dabaver@wsu.ac.za; Tel.:+ 27-47-502-2052 or +27-74-768-5149; 2 Division of Medical Microbiology, Department of Laboratory Medicine and Pathology, New Medical School, Walter Sisulu University, Nelson Mandela Drive, Mthatha 5100, Eastern Cape, South Africa

**Keywords:** prevalence, geo-mapping, schistosomiasis, Eastern Cape

## Abstract

Schistosomiasis is caused by infection with trematode flukes of the genus Schistosoma. More than 700 million people worldwide are estimated to be susceptible to infection. In sub-Saharan Africa, schistosomiasis is the second most widespread neglected tropical disease after malaria. This retrospective investigation evaluated the incidence and impacts of schistosomiasis on communities across three major districts of the Eastern Cape province in South Africa using a cross-sectional retrospective observational analysis of secondary data from patients with microscopically confirmed schistosomiasis between 2019 and 2020. This study focused upon both rural and semi-urban areas, including Bizana, Butterworth, Centane, Elliotdale, Flagstaff, Idutywa, Lusikisiki, Libode, Mqanduli, Port St. Johns, Willowvale, and Mthatha. Data were obtained from three districts—Alfred Nzo, Amatole, and OR Tambo—covering both rural and semi-urban regions. This study included patients of all ages who submitted urine samples for schistosomiasis testing in the specified districts. A simple random sampling method was used to select 337 clinical records from the National Health Laboratory Service (NHLS) of Mthatha. Hospital records from the NHLS Microbiology Department of Mthatha were analyzed. St Barnabas Laboratory had the highest frequency of cases (34.1%), followed by Greenville Depot (17.8%) and Willowvale Laboratory (11.3%). Most cases were in the 10–19 age group (63.4%), followed by those under 10 years of age (24.9%). Male patients constituted 76.4% of the cases, while female patients accounted for 23.6%. Viable ova were observed in 98.2% of the samples. This study highlights a significant prevalence of schistosomiasis in the Eastern Cape province, with a higher incidence in rural areas and among males aged 10–19. These findings underscore the need for targeted public health interventions and continuous monitoring to control and prevent schistosomiasis in this region.

## 1. Introduction

Schistosomiasis, also known as Bilharzia, is an infectious disease caused by trematode flukes of the genus *Schistosoma* [1]. Recognized as a neglected tropical disease (NTD), it is estimated that approximately 732 million people worldwide are susceptible to this infection [2]. In sub-Saharan Africa, schistosomiasis is the second most widespread NTD after malaria [3]. The African continent is affected by four *Schistosoma* species: *Schistosoma mansoni* (intestinal), *Schistosoma haematobium* (urogenital), *Schistosoma intercalatum* (intestinal), and *Schistosoma guineensis* (intestinal) [4]. Among these, *S. mansoni* and *S. haematobium* are the most prevalent and widespread, while *S. intercalatum* and *S. guineensis* are rare and confined to Central African countries [5].

Humans contract schistosomiasis through contact with freshwater contaminated with skin-penetrating cercariae [3]. Acute infections can cause symptoms such as malaise, skin rashes, fever, and abdominal pain, while chronic infections are associated with liver, lung, intestinal, or urogenital diseases, depending on the *Schistosoma* species involved [6]. Prolonged exposure to the infection can lead to severe complications, including bladder cancer, pulmonary hypertension, and urinary tract obstructions, potentially resulting in death. In endemic regions, schistosomiasis predominantly affects children aged 5 to 15 years and populations living near water bodies [6]. Factors contributing to the high risk among school-aged children include poor hygiene, frequent exposure to contaminated water, and low levels of education. The disease is more prevalent in impoverished rural communities, particularly in areas where fishing and agriculture are common. Daily activities such as washing clothes and fetching water from infected rivers expose women and children to the infection, while recreational activities like swimming in contaminated water bodies increase children’s vulnerability [2]. Schistosomiasis negatively impacts child development, pregnancy outcomes, and agricultural productivity, perpetuating poverty among approximately 500 million inhabitants of sub-Saharan Africa [3].

Diagnosing schistosomiasis involves the microscopic examination of stool and urine samples to detect *Schistosoma* eggs, which are identifiable by their distinctive size, shape, and lateral spine [7]. Viable ova of this parasite have a fully intact shell, which serves as a protective layer for the developing larva (miracidium) inside [8]. The shell appears smooth and unbroken, with no signs of degeneration or collapse. *S. haematobium* ova are typically oval or elongated with a characteristic terminal spine at one end [9]. They measure about 110–170 µm in length and 40–70 µm in width. The terminal spine is a distinguishing feature for identifying *S. haematobium* ova under a microscope [10]. Inside a viable ovum, the developing miracidium (the larval form of the parasite) may show signs of movement, which indicates viability [11]. The cytoplasm within viable eggs appears granular and well organized, supporting the developing larva [8]. Viable eggs often have a translucent appearance, allowing visibility of internal structures and larval movement, compared to non-viable ova, which may appear darker or opaque due to cellular breakdown [12].

In schistosomiasis, secondary bacterial infections can complicate the disease, prompting the use of antibiotics as a supportive treatment. However, the frequent use of antibiotics raises concerns about potential antibiotic resistance. Resistance is a growing issue in endemic regions, where access to healthcare and controlled antibiotic use may be limited, increasing the risk of resistance among bacteria associated with schistosomiasis infections [13,14].

Additionally, red and white blood cells play significant roles in the body’s response to *Schistosoma* infection. *Schistosoma* eggs trapped in tissues can elicit a strong immune response. This response may lead to tissue damage and the release of hemoglobin, altering red blood cell counts [15]. White blood cell activity is a hallmark of schistosomiasis, reflecting the body’s attempt to control and mitigate parasite-induced damage [14].

Preventative measures include providing access to clean tap water, preventing sewage contamination of freshwater, and removing intermediate host snails [1]. Praziquantel is an effective treatment, curing 75–85% of *S. haematobium* and 65–85% of *S. mansoni* infections [16]. This study aims to evaluate the negative impacts of schistosomiasis on the community and compare its incidence across the three major districts of the Eastern Cape province.

## 2. Methods

### 2.1. Study Design and Setting

A cross-sectional retrospective study was conducted using secondary data from patients with microscopically confirmed schistosomiasis from urine samples collected between 2019 and 2021. This study covered three districts in the Eastern Cape province of South Africa: Alfred Nzo, Amatole, and OR Tambo. Both rural and semi-urban areas were included, with rural areas comprising Bizana, Butterworth, Centane, Elliotdale, Flagstaff, Idutywa, Lusikisiki, Libode, Mqanduli, Port St. Johns, and Willowvale, and the semi-urban area being Mthatha.

### 2.2. Population and Sampling

The study population included patients of all ages who submitted urine samples for schistosomiasis testing at various laboratories within hospitals in the specified districts of Alfred Nzo, Amatole, and OR Tambo. The urine samples were forwarded to the National Health Laboratory Service (NHLS) of South Africa, a central agency which plays a significant role in providing laboratory services for government hospitals and clinics. The NHLS operates as a national entity under the South African Department of Health, managing and standardizing laboratory services across government healthcare facilities. When patients in government hospitals require laboratory tests (such as blood tests, tissue samples, or infectious disease screenings), samples are collected at the hospital or clinic level. The secondary data used for this study were drawn from the clinical records of patients, which were sampled from the NHLS database in Mthatha, Eastern Cape. After ethical approval (protocol number 129/2021), a simple random sampling method was used to select 337 clinical records from the NHLS of Mthatha. There were no exclusion criteria, and patients of all ages and genders during the study period were included. The data include demographic characteristics of the participants, the geographic distribution of the disease, leucocytes, erythrocytes, epithelial cells, casts, crystals, parasite, and bacterial culture results. Relevant data points from these records were carefully reviewed and recorded in an Excel spreadsheet to create a structured dataset for statistical analysis. This approach facilitates efficient data handling, organization, and preparation for subsequent statistical analysis, ensuring that the extracted data are standardized, accessible, and ready for analytical processing.

### 2.3. Data Analysis

Hospital records from the National Health Laboratory Services (NHLS) Microbiology Department of Mthatha were analyzed using IBM SPSS version 20 (IBM Corporation, 1 New Orchard Road, Armonk, NY, USA). Statistical tests, including Fisher’s exact test, Pearson’s Chi-squared test, and Wilcoxon’s rank sum test, were employed to compare demographic variables. The Shapiro–Wilk test was used to assess the normality of quantitative data. Descriptive statistics were used to summarize the data, and the results were presented in the form of graphs and tables.

### 2.4. Ethical Considerations

Although the research used secondary data with no risk to patients, all due ethical processes were observed. This study was conducted in accordance with the Declaration of Helsinki and was approved by the Institutional Review Board of the Postgraduate Education, Training, and Research Ethics Committee, Faculty of Medicine and Health Sciences, Walter Sisulu University (protocol code 129/2021, dated 8 November 2021).

## 3. Results

A total of 337 *Schistosoma* spp.-positive patient records were obtained from the National Health Laboratory Service (NHLS) of Mthatha, Microbiology Department. This study encompassed patients of all ages from three districts in the Eastern Cape: Alfred Nzo, Amatole, and OR Tambo. Detailed demographic information is presented in Table 1.

### Demographic Characteristics

The demographic characteristics of the study population are summarized in Table 1. Age distribution showed the highest prevalence in the 10–19 age group (61.1%), followed by the 0–9 age group (24%), while the age group of patients aged 20 and above was (14.8%).

Of the study population, 33.3% had a positive bacterial culture, and 20.6% had a negative bacterial culture. The remaining 47% had unavailable, inconclusive, or unprocessed bacterial culture results, which were therefore not included in the positive or negative outcome categories.

The median age of female patients was 14 years (IQR: 11.0–19.2), while the median age of male patients was 12 years (IQR: 10.0–14.0), with a statistically significant difference (*p* = 0.013). The age-specific prevalence rates showed significant differences between genders in the 0–9 (*p* = 0.003) and 20–29 (*p* = 0.003) age groups. The geographic distribution analysis indicated no significant difference in prevalence between genders in OR Tambo (*p* = 0.11) and semi-urban areas (*p* = 0.4).

About 98% of the samples had eggs (ova) of the parasite that were alive, with the potential to develop further and infect the next host during their life cycle if they encountered appropriate environmental conditions (Table 2).

**Figure 1:**
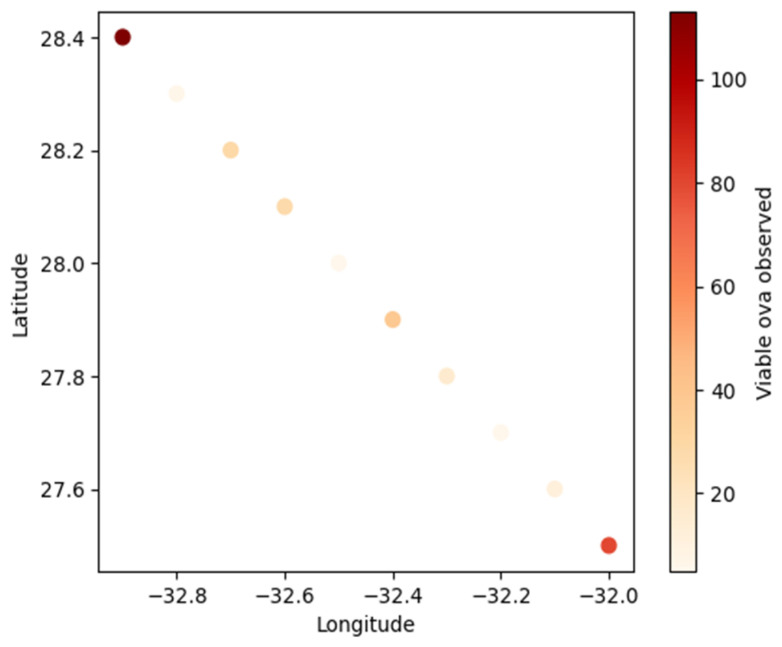
Geographic distribution of viable ova observed across locations, with longitude and latitude coordinates indicating the sampling sites.

**Figure 2:**
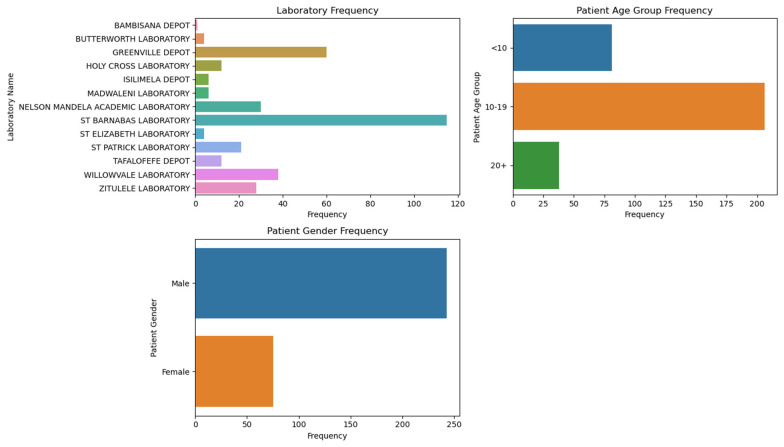
Geo-mapping of *Schistosoma haematobium* in the Eastern Cape of South Africa.

Figure 1 shows geographic distribution of viable ova observed across locations, with longitude and latitude coordinates indicating the sampling sites. The geographic distribution of schistosomiasis cases revealed that the majority were from the OR Tambo district (73.3%, 95% CI: 65.9–80.7), followed by Amatole (20.7%, 95% CI: 13.8–27.5) and Alfred Nzo (6%, 95% CI: 1.9–10.1). The laboratory frequencies show that St Barnabas Laboratory had the highest frequency of cases (34.1%), followed by Greenville Depot (17.8%) and Willowvale Laboratory (11.3%) (Figure 2).

## 4. Discussion

Schistosomiasis remains a significant public health challenge in tropical and subtropical regions, driven by environmental, socio-economic, and behavioral factors. Transmission is sustained by freshwater bodies that harbor intermediate snail hosts, particularly in regions like sub-Saharan Africa [17] with abundant lakes, rivers, and irrigation systems. Seasonal variations and water management practices further influence prevalence, with heightened transmission during the rainy season when human–water contact increases [18].

In the Eastern Cape province of South Africa, schistosomiasis is particularly prevalent in rural areas due to limited access to clean water and sanitation. School-aged children and young adults are disproportionately affected due to frequent water contact during activities like swimming and fishing. Women involved in domestic chores, such as washing clothes, also face significant exposure, with female genital schistosomiasis posing a notable concern [19].

Socio-economic inequities exacerbate the burden, particularly in impoverished rural communities with inadequate healthcare access. Delayed diagnosis and treatment contribute to disease persistence [20,21], while occupational exposure in agriculture, fishing, and irrigation further increases risk [22]. Addressing these inequities is essential for disease control.

Behavioral factors and knowledge gaps also drive transmission. Misconceptions and limited awareness about schistosomiasis hinder prevention, underscoring the need for tailored health education programs to promote protective behaviors and timely medical intervention.

The predominance of *Schistosoma haematobium* infections was confirmed through diagnostic findings, including high rates of ova, hematuria, and pyuria in urine analysis. Inflammatory markers, such as elevated leucocyte and erythrocyte counts, and evidence of renal and urinary tract pathology [23,24], including epithelial cells and casts, were also noted. The presence of crystals and yeast indicated secondary infections or metabolic disturbances.

Co-infections with bacteria, observed in 33.3% of cases, highlight the need for integrated management strategies. Antimicrobial substances detected in nearly half of the samples point to bacterial involvement, emphasizing the importance of bacterial cultures and sensitivity testing to guide treatment. Addressing antimicrobial resistance is critical to mitigating morbidity and improving outcomes [22,23].

Demographic analysis showed a higher prevalence among male (78.7%) than female patients (21.3%), consistent with findings from other regions [25,26]. The most affected age group featured people between10 and 19 years of age, reflecting increased recreational and occupational water exposure [27]. Geographically, the OR Tambo district accounted for most of the cases (73.3%), followed by the Amatole and Alfred Nzo districts, illustrating the combined impact of environmental and socio-economic factors on disease distribution [28].

## 5. Conclusions

The findings underscore the need for targeted public health interventions to address schistosomiasis in endemic regions. Improving access to clean water and sanitation facilities is paramount, alongside routine screening programs to detect and treat infections early. Community-focused health education campaigns are essential to raise awareness and encourage behaviors that reduce exposure. Integrated treatment approaches that address both schistosomiasis and bacterial co-infections can mitigate the disease burden effectively.

Future research should prioritize exploring environmental risk factors, the efficacy of interventions, and the relationship between schistosomiasis and other health outcomes, such as anemia. These efforts will provide additional insights to guide comprehensive strategies for controlling and eventually eliminating schistosomiasis in the Eastern Cape and similar endemic regions.

## Figures and Tables

**Table 1:** Demographic characteristics of the study population.

Category	Subcategory	Frequency	Valid Percent
Patient Age Group	<10	81	24.9
	10–19	206	63.4
	20+	50	11.7
Patient Gender	Male	243	76.4
	Female	94	23.6

**Table 2:** Summary of diagnostic and epidemiological data for schistosomiasis in the Eastern Cape province.

Category	Subcategory	Frequency	Percent	Valid Percent
Sample Type	Urine	333	98.8	
	Blood	1	0.3	
	Both Blood and Urine	3	0.9	
Leucocytes	<750,000	118		65.9
	750,000+	61		34.1
Erythrocytes	<750,000	58		31.7
	750,000+	125		68.3
Epithelial cells	1+	65		34.6
	2+ (Moderate)	15		8
	3+ (Numerous)	9		4.8
	Not Observed	99		52.7
Casts	Casts Present	45		23.9
	Not Observed	143		76.1
Crystals	Crystals Present	14	4.2	100
	Missing	323	95.8	
Parasite (adult *S. haematobium*)	Parasite Present	8		88.9
	Not Observed	1		11.1
*Schistosoma haematobium* ova	Viable ova observed	331	98.2	
	No ova observed	6	1.8	
Antimicrobial substances	Present	117		46.4
	Absent	135		53.6
Erythrocytes during Ova Exam	1+	12		5.3
	2+ (Moderate)	19		8.4
	3+ (Numerous)	193		85.4
	Not Observed	2		0.9
Sensitivity Status	Sensitive to one Antibiotic	40		87
	Sensitive to two Antibiotics	4		8.7
	Sensitive to three Antibiotics	2		4.3

## Data Availability

The data that support the findings of this study are not publicly available due to privacy and ethical restrictions. The data contain sensitive information that could compromise the privacy of the research participants. As such, access to these data is restricted and can only be provided upon reasonable request to the corresponding author, subject to approval by the relevant ethics review board.
